# MEEAFusion: Multi-Scale Edge Enhancement and Joint Attention Mechanism Based Infrared and Visible Image Fusion

**DOI:** 10.3390/s24175860

**Published:** 2024-09-09

**Authors:** Yingjiang Xie, Zhennan Fei, Da Deng, Lingshuai Meng, Fu Niu, Jinggong Sun

**Affiliations:** Systems Engineering Institute, Academy of Military Sciences, PLA, Beijing 100166, China; xieyj2107@163.com (Y.X.); fzn41240200@163.com (Z.F.); deng_da_jx@163.com (D.D.); mengls1989@sina.com (L.M.)

**Keywords:** edge enhancement, attention mechanism, image fusion, infrared image, visible image

## Abstract

Infrared and visible image fusion can integrate rich edge details and salient infrared targets, resulting in high-quality images suitable for advanced tasks. However, most available algorithms struggle to fully extract detailed features and overlook the interaction of complementary features across different modal images during the feature fusion process. To address this gap, this study presents a novel fusion method based on multi-scale edge enhancement and a joint attention mechanism (MEEAFusion). Initially, convolution kernels of varying scales were utilized to obtain shallow features with multiple receptive fields unique to the source image. Subsequently, a multi-scale gradient residual block (MGRB) was developed to capture the high-level semantic information and low-level edge texture information of the image, enhancing the representation of fine-grained features. Then, the complementary feature between infrared and visible images was defined, and a cross-transfer attention fusion block (CAFB) was devised with joint spatial attention and channel attention to refine the critical supplemental information. This allowed the network to obtain fused features that were rich in both common and complementary information, thus realizing feature interaction and pre-fusion. Lastly, the features were reconstructed to obtain the fused image. Extensive experiments on three benchmark datasets demonstrated that the MEEAFusion proposed in this research has considerable strengths in terms of rich texture details, significant infrared targets, and distinct edge contours, and it achieves superior fusion performance.

## 1. Introduction

Image fusion, as a subset of information fusion, involves analyzing and fusing image data from an identical scene acquired by multiple sensors to create more informative fused images. In general, infrared sensors like infrared cameras are suitable for harsh environments and occlusion circumstances such as darkness, rain, and fog. However, infrared images bear the shortcomings of low contrast and resolution, as well as lacking detailed texture. Additionally, they are susceptible to interference from background noise [1]. Visible light sensors like optical cameras record images more closely aligned with human vision, with higher resolution and more detailed texture. Notably, the imaging quality is inferior under low light and occlusion conditions [2]. Therefore, complementing the advantages between these sensors and fusing visible and infrared images enables the resulting images to include both texture details and infrared target information. Embedding infrared and visible image fusion (IVIF) algorithms on carriers equipped with infrared and visible light sensors can assist in realizing tasks such as personnel search and rescue, autonomous driving, remote sensing monitoring, and defense reconnaissance. Moreover, integrating IVIF with advanced vision tasks such as target detection, tracking, and semantic segmentation can facilitate the performance improvement of these tasks. Therefore, IVIF has become a research focus in recent years.

The process of IVIF mainly comprises two steps: information extraction and information fusion. The challenge lies in how to achieve the maximum extraction of features and information from the image and integrate them naturally, to acquire high-quality images with complementary information. The available fusion algorithms are divided into two types: traditional methods and deep learning (DL)-based methods. Traditional approaches primarily include multi-scale transform [3,4], sparse representation [5], and subspace [6]. These methods rely on pyramidal and wavelet transform techniques, overcomplete dictionaries, and principal and secondary component analysis to implement image decomposition and fusion. Traditional fusion methods provide strong interpretability but hinge on manually designing feature extraction and fusion rules. The computation is complex and time-consuming, and the fusion results frequently show texture blurring, poor contrast, and artifacts. Benefiting from the breakthrough of computational power resources, DL technology has rapidly evolved and been introduced into the field of image fusion. This facilitates the automatic training of model parameters, thereby minimizing the influence of human factors. As a result, the ability of the network to extract complementary image information is optimized, realizing simple yet efficient fusion. Since Liu et al. [7] introduced CNN into the multi-focus image fusion task, numerous DL-based fusion methods have been developed, including autoencoder (AE)-, CNN-, GAN-, and task-driven-based algorithms.

**Figure 1:**
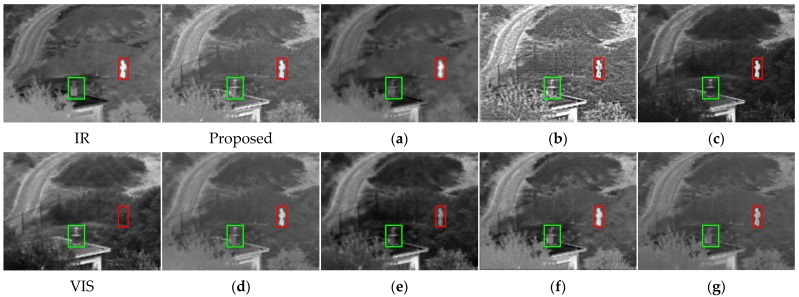
Display of fusion results. IR and VIS denote infrared image and visible image, and Figures (**a**–**g**) show the fusion results of FusionGAN [8], IPLF [9], STDFusionNet [10], DenseFuse [11], RFN-Nest [12], PMGI [13], and FLFuse-Net [14], respectively. The red and green boxes outline the salient targets and detail regions.

Some current IVIF algorithms suffer from inadequate extraction of source image detail features or loss of global features, resulting in distorted fusion images, unclear edge outlines, and artifacts (as demonstrated in Figure 1a,b). Some approaches prioritize retaining the textural details from visible images and infrared salient objects in images and employ manually created masks to achieve clear infrared targets. However, such approaches are time-consuming and laborious and suffer from inadequate labeled data. The limited feature-fitting capacity of the fusion network causes the loss of infrared or visible information, leading to darker or brighter images (as illustrated in Figure 1c). Regarding the feature fusion strategy, some AE networks necessitate the manual design of rules when fusing image features, which brings in subjective factors and makes it challenging to determine the optimal fusion strategy, leaving the fusion quality poor (as illustrated in Figure 1d,e). Most IVIF methods overlook the significance of intermediate layer features and solely focus on fusing the final depth features. They ignore the complementarity of information between the source images, ultimately leading to feature degradation. Even if partial end-to-end fusion algorithms accomplish feature interaction in the middle layer, they just perform simple convolution operations upon the features from the dual branches. Insufficient supplementary information mining leads to fusion results with unclear texture details and insignificant infrared targets (as displayed in Figure 1f,g).

In response to the aforementioned shortcomings, this work develops a multi-scale edge enhancement and joint attention mechanism-based IVIF method (MEEAFusion). MEEAFusion utilizes convolution kernels of varying scales to fully extract the shallow feature information from the original image. Subsequently, it devises a multi-scale gradient residual block (MGRB) and a cross-transfer attention fusion block (CAFB) to enhance the edge contours of deep feature representations, while realizing feature interactions and pre-fusion. Ultimately, the shallow and last deep features are merged to reconstruct the image without manually designing any fusion rules.

Contributions to this paper are summarized below.

(1) An end-to-end IVIF model is provided, primarily composed of a shallow multi-scale feature extraction module, a multi-scale gradient residual block (MGRB), and a cross-transfer attention fusion block (CAFB). Dense connections are also incorporated to alleviate the information loss caused by the feature flow.

(2) MGRB is innovatively constructed by integrating several scales of gradient convolution, which could fully extract the textural details and semantic information from the image and obtain the depth features with enhanced edge contour. The MGRB module enables further promotion of the detailed description capability of the network.

(3) CAFB is created by incorporating the spatial and channel attention mechanism to achieve deep feature interaction between two paths, obtaining pre-fused features enriched with complementary information and common features from the two source images. The CAFB module abandons the traditional mindset of extracting first and fusing later and realizes the parallel processing of feature extraction, interaction, and fusion.

(4) Experiments on three generalized IVIF datasets demonstrate that the fusion results produced by the proposed method have abundant texture details, prominent infrared targets, and sharp edge contours, and they outperform those of mainstream fusion approaches in terms of subjective and objective evaluation metrics. It offers a novel, structurally simple yet high-performance solution for complementary multimodal image fusion.

## 2. Related Work

### 2.1. DL-Based IVIF Methods

The AE-based method is the most classical approach among the DL-based IVIF, and the essential idea is consistent with that of traditional approaches. The encoder first obtains the feature from the original image via a feature extraction network, followed by feature information fusion according to pre-established fusion strategies. Eventually, the decoder yields a fused image by reconstructing the fused features [15,16]. DeepFuse [17] performs the same convolution operation on a set of overexposed and underexposed images to extract the feature maps, which are then summed and fused to rebuild the image. This strategy only focuses on the last layer of features and ignores the usage of information from the intermediate layer of features. The DenseFuse [11] network introduces densely connected blocks to extract multi-layer depth information without losing information from the intermediate layer, considerably boosting the fused image quality. NestFuse [18] utilizes Nest connections to decode the fused features at several scales, which could increase the multi-scale representation of the image features. RFN-Nest [12] replaces the fusion strategy of NestFuse with residual networks to fuse the features at different scales, resulting in the fused image containing more detailed information. An atrous spatial pyramid network with different expansion rates is utilized by EDAfuse [19] to extract depth features with different scales, and the fused image can contain more details and salient object characters. FPN, as an encoding network, can fully extract and fuse features from multiple scales and levels in two images, resulting in methods like FPNFuse [20] and PG-Fusion [21].

CNN-based fusion methods eliminate the need to construct fusion rules to accomplish end-to-end fusion. Liu et al. [7] first completed the multi-focus image fusion task with the help of CNN. PMGI [13] views image fusion as an issue of preserving intensity and gradient information. To this end, the method employs two identical paths to extract the gradient information and intensity information and introduces dense connections to reuse the intermediate layer features to prevent information loss. Additionally, a cross-path exchange module is developed to pre-fuse the features and strengthen the information interaction. FLFuse-Net [14] realizes fast fusion with the fully convolutional network and designs a significant information edge compensation branch between the infrared image and the decoder, which is used to retrieve the edge information of the significant infrared target to sharpen the fused image contour. U2fusion [22] combines information measurement and adaptive preservation strategies, making it suitable for multiple image fusion tasks. DRSNFuse [23] adopts deep residual blocks to extract features and further separates the base and detail parts from the feature map. Finally, the shallow features extracted by the base, detail, and residual blocks are integrated and reconstructed to yield the fused image.

IVIF typically lacks standard labels available, and GAN is particularly suitable for such unsupervised fusion tasks. The advantage lies in its reliance on the confrontation between the discriminator and the generator to optimize the generative capacity for creating the desired fused image. FusionGAN [8] innovatively introduces GAN to the IVIF field. Without base labeling, the discriminator is trained with the visible image as the true value, and the generator is prompted to produce an image containing more visible image details. Nevertheless, an individual discriminator might lead to algorithm collapse, meaning that the fused results are biased toward infrared or visible images. Consequently, DDcGAN [24] proposed a dual discriminative conditional GAN that leverages two discriminators to ensure that the fused image retains both the thermal object information and detailed texture from the infrared image and visible image, thereby improving the robustness of the fusion network and maintains the information balanced among the two images. The generator of DUGAN [25] integrates information from image content and gradient, while the discriminator uses a U-shaped architecture to drive the fused image to incorporate richer detailed features and global information.

The union of image fusion and downstream tasks as a whole can guide and promote each other to achieve well-fused results. SeAFusion [26] combines IVIF with high-level semantic segmentation tasks and feeds the fusion network output into the segmentation network to evaluate the fusion quality with the segmentation outcomes. Furthermore, a joint training strategy was developed, and semantic loss and content loss were proposed to simultaneously guide and optimize fusion network and segmentation network training. Zhang et al. [27] proposed a real-time fusion approach employing an adaptive weighting strategy to trade off the speed and quality of IVIF on an embedded platform, which joins image fusion with downstream target detection to attain faster fusion speeds and higher detection accuracies. IRFS [28] developed a joint paradigm of image fusion and advanced vision tasks, prioritizing image fusion as the primary objective while incorporating multimodal salient target detection as a subsidiary task. This method aims to facilitate saliency-guided image fusion, and a cross-loop training strategy is designed to aid in training network parameters. RSDFusion [29] incorporates IVIF and semantic segmentation to achieve the real-time fusion effect, successfully preserving the detailed textures and remarkable objects in the source image.

Moreover, recently, researchers have introduced the latest transformer technique [30,31,32], mamba model [33], and diffusion model [34,35] to the multimodal image fusion task and achieved favorable fusion results as well. A growing number of novel algorithms will emerge and develop in the IVIF field.

### 2.2. Attention Mechanism

The attention mechanism imitates the human visual focusing mechanism by assigning different weights to various targets, reflecting the level of relevance of the information. It has been broadly applied in computer vision and has gradually been introduced to the IVIF problem, yielding a series of advanced fusion algorithms.

Xu et al. [36] presented an IVIF algorithm based on the CBAM [37] module with different scale kernels to extract multi-scale feature maps from both spatial and channel dimensions. RDCa-Net [38] employs channel attention to focus on salient features across different feature layers and adopts a self-attention mechanism to concern contextual information. This approach adaptively obtains the weight parameters while calculating the loss function weights, allowing the fused result to retain more detailed features. Zhan et al. [39] embedded a global attention mechanism into the fusion algorithm based on a semantic segmentation task to capture the contextual dependencies over a long distance, thus adjusting the channel weights. AttentionFGAN [40] introduces the attention mechanism into GAN-based methods to allow the network to focus more on the texture details of visible images and prominent targets of infrared images. Cross-modal attention [41] is suitable for the IVIF task due to the ability to notice the information differences between different modalities. CrossFuse [42] devised a special cross-attention module to enhance the mutual information across multimodal images, attaining superior fusion performance. 

Although the existing algorithms can focus more on crucial region information in the source image, they neglect the complementary features between different modalities, resulting in insufficient information exchange. Therefore, this paper designs the CAFB module to capture complementary information across multiple modalities adequately.

## 3. Method

This section presents a novel IVIF method, MEEAFusion, and provides a detailed description of the design and composition of each module and loss function.

### 3.1. Network Framework

**Figure 2:**
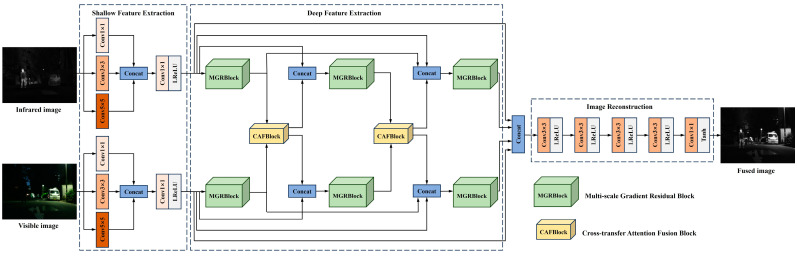
MEEAFusion—overall framework.

As illustrated in Figure 2, the innovative MEEAFusion algorithm in this study first focuses on the feature heterogeneity between source images. In the shallow feature extraction stage, the convolutional layers of infrared and visible image paths are trained independently to generate feature maps containing the unique characteristics of each source image. To avoid the fixed receptive fields and potential loss of feature information caused by single convolution, the method utilizes convolution kernels of different scales to implement the feature extraction process. This ensures that the generated feature map covers a wide range of receptive fields, thereby enhancing the diversity of image information.

Then, in the deep feature extraction stage, a multi-scale gradient residual block (MGRB) is innovatively constructed, which consists of different scales of Sobel gradient operators and residual connections. The MGRB module is aimed at optimizing image semantic information while strengthening feature edges and details. To eliminate the information loss caused by manual intervention or fusing features only before reconstructing the image, MEEAFusion designs the cross-transfer attention fusion block (CAFB). This module enables the facilitation of feature interaction between source images, meanwhile carrying out complementary feature cross-transfer between the two branches. Thus, each branch has access to the complementary information of the other branch in advance, i.e., pre-fusion. During feature extraction, the MGRB is densely connected to thoroughly convey different layers of feature information without adding any parameter burden to prevent information loss of deep features when the network goes deep. Under continuous feature extraction, interaction, and transmission, the feature information of visible and infrared images is sufficiently integrated and unified. A completely symmetric network structure with shared parameters is adopted for the deep feature extraction to train the convolutional kernel adapted to the feature extraction of both visible and infrared images and to further lower the network parameters.

Finally, the shallow and last deep feature results from the two paths are concatenated as inputs to the image reconstruction module to maximize the use of feature data and alleviate feature loss. Four consecutive 3 × 3 convolutions and one 1 × 1 convolution are utilized to reconstruct the features to obtain the ultimate fused image. Apart from the last convolution, whose activation function is Tanh, all the other convolution operations adopt Leaky Rectified Linear Unit (LRelu) as their activation functions.

In addition, pooling, upsampling, and downsampling operations are not employed throughout the fusion network, as they are prone to information loss and may also introduce noise. MEEAFusion adopts padding to always ensure that the image size remains constant during feature extraction and reconstruction. A detailed description of the design and components of each module is given below.

#### 3.1.1. Shallow Multi-Scale Feature Extraction Module

Most fusion networks use a series of 3 × 3 convolutional kernels for feature extraction on the input image, resulting in a fixed receptive field. By contrast, using convolutional kernels of varying scales can obtain feature maps with different receptive fields, which have rich local and contextual information and can provide high-quality feature information for subsequent deep feature extraction.

For the given infrared image $I_{ir}$ and visible image $I_{vis}$, this paper uses three scales of convolution kernel 1 × 1, 3 × 3, and 5 × 5 to extract image feature information. The resulting features are concatenated; then, the channel number is reduced by 1 × 1 convolution, and the source image shallow features are obtained after activated by the LRelu function. The output features are calculated as follows:

(1) $$F_{ir,vis}=\sigma \left(Conv_{1}\left(C\left(Conv_{1}I_{ir,vis},Conv_{3}I_{ir,vis},Conv_{5}I_{ir,vis}\right)\right)\right)$$

where $Conv_{1}$, $Conv_{3}$, and $Conv_{5}$ denote 1 × 1, 3 × 3, and 5 × 5 convolutions, C(∙) denotes channel concatenation, and σ(∙) stands for the activation function LReLU. $F_{ir}$ and $F_{vis}$ denote the shallow features of the infrared image and visible image.

#### 3.1.2. Multi-Scale Gradient Residual Block (MGRB)

**Figure 3:**
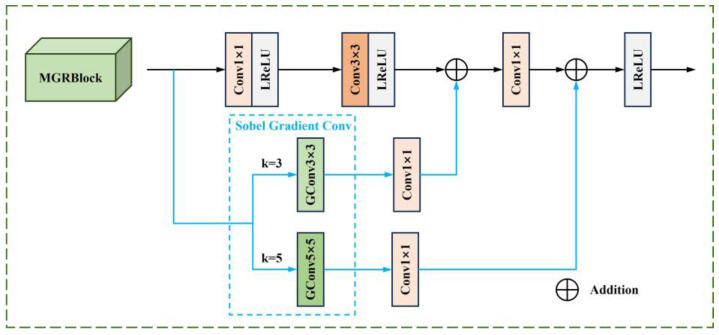
MGRB module structure.

The MGRB module is designed by combining different scales of gradient operators containing one main stream and two residual gradient streams. The detailed structure is presented in Figure 3.

Conventional convolutional operations are performed on the mainstream to extract feature semantic information, and efficient Sobel gradient convolution is employed on the branch to improve the fine grain of the features. Two branches utilize convolution kernels with k = 3 and k = 5 for the extraction of edge information. When given the input feature $F_{ir,vis}$, the outputs of the gradient convolution with different scales are

(2) $$G_{3\times 3}F_{ir,vis}=\left|G_{3\times 3vertical}F_{ir,vis}\right|+\left|G_{3\times 3horizontal}F_{ir,vis}\right|$$

(3) $$G_{5\times 5}F_{ir,vis}=\left|G_{5\times 5vertical}F_{ir,vis}\right|+\left|G_{5\times 5horizontal}F_{ir,vis}\right|$$

where $G_{3\times 3horizontal}$, $G_{3\times 3vertical}$, $G_{5\times 5horizontal}$, and $G_{5\times 5vertical}$ represent the 3 × 3 and 5 × 5 gradient convolution components in the horizontal and vertical directions, respectively, and the specific convolution kernel parameters are as follows:

(4) $$G_{3\times 3vertical}=\frac{1}{9}\left[\begin{array}{ccc} -1 & 0 & +1\\ -2 & 0 & +2\\ -1 & 0 & +1 \end{array}\right],\;G_{3\times 3horizontal}=\frac{1}{9}\left[\begin{array}{ccc} +1 & +2 & +1\\ 0 & 0 & 0\\ -1 & -2 & -1 \end{array}\right]$$

(5) $$G_{5\times 5vertical}=\frac{1}{25}\left[\begin{array}{ccccc} -1 & -2 & 0 & +2 & +1\\ -4 & -8 & 0 & +8 & +4\\ -6 & -12 & 0 & +12 & +6\\ -4 & -8 & 0 & +8 & +4\\ -1 & -2 & 0 & +2 & +1 \end{array}\right],\;G_{5\times 5horizontal}=\frac{1}{25}\left[\begin{array}{ccccc} +1 & +4 & +6 & +4 & +1\\ +2 & +8 & +12 & +8 & +2\\ 0 & 0 & 0 & 0 & 0\\ -2 & -8 & -12 & -8 & -2\\ -1 & -4 & -6 & -4 & -1 \end{array}\right]$$

The 1 × 1 convolution of the branch allows for adjusting the channel dimension of the feature map to ensure consistency with that of the mainstream. Finally, the element-wise addition is performed to map the gradient features of the residual stream onto the mainstream features, thereby realizing edge compensation of the depth feature. Thus, for the MGRB module, in the case of a given input feature $F_{ir,vis}$, the output ${F^{\prime }}_{ir,vis}$ is calculated as follows:

(6) $${F^{\prime }}_{ir,vis}=\sigma \left(Conv_{1}\left(Conv_{1,3}F_{ir,vis}⊕Conv_{1}\left(G_{3\times 3}F_{ir,vis}\right)\right)⊕Conv_{1}\left(G_{5\times 5}F_{ir,vis}\right)\right)$$

where $Conv_{1,3}$ indicates that the $Conv_{1}$ + LReLU and $Conv_{3}$ + LReLU operations are performed sequentially.

**Figure 4:**
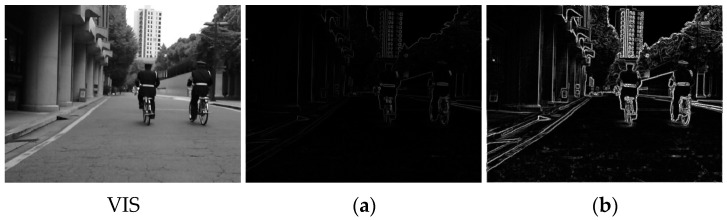
Gradient convolution results of the visible image. (**a**,**b**) show the 3 × 3 and 5 × 5 Sobel convolution results, respectively.

Figure 4 specifically demonstrates the two gradient convolution results. The 3 × 3 gradient convolution roughly captures the image edge information, while the 5 × 5 gradient convolution has a wider range of receptive fields and could extract clearer texture details, such as the contours of the human and the tree branches. In particular, the texture inside the tree trunk is notably outlined as well, which can hardly be distinguished by the eye. Thus, using gradient operators of different scales can acquire richer texture information and strengthen the fused image edge details. Replacing the regular stacked convolution with the MGRB module can further enhance the fine-grain feature and the ability of the network to describe the details.

#### 3.1.3. Cross-Transfer Attention Fusion Block (CAFB)

**Figure 5:**
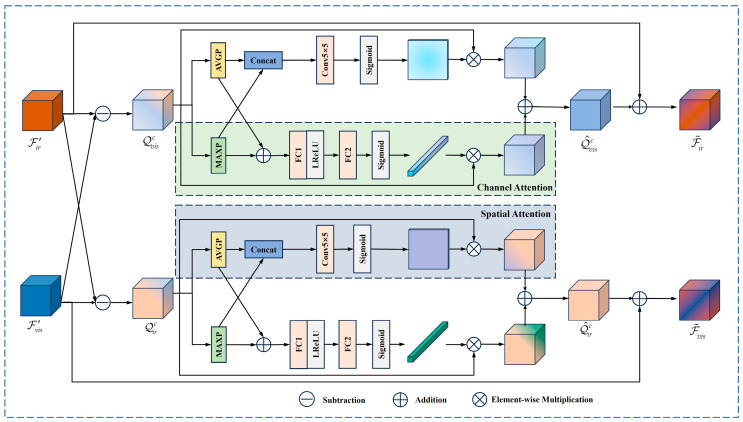
CAFB module structure.

CAFB works in the procedure of deep feature extraction, with a refined workflow as follows: firstly, it obtains the complementary features between the two source image features. Then, spatial attention (SA) and channel attention (CA) are utilized jointly to assign weights to both complementary information, effectively suppressing the irrelevant features in spatial and channel dimensions. Finally, the weighted complementary feature maps are incorporated into the input features to realize the feature information pre-fusion. Figure 5 exhibits the particular architecture of CAFB.

Specifically, the differential idea in [43] is adopted; i.e., the two source image features can be reformulated as

(7) $$\begin{array}{l} {F^{\prime }}_{ir}=\frac{{F^{\prime }}_{ir}+{F^{\prime }}_{vis}}{2}+\frac{{F^{\prime }}_{ir}-{F^{\prime }}_{vis}}{2}\\ {F^{\prime }}_{vis}=\frac{{F^{\prime }}_{vis}+{F^{\prime }}_{ir}}{2}+\frac{{F^{\prime }}_{vis}-{F^{\prime }}_{ir}}{2} \end{array}$$

Therefore, the two modal features can be regarded as an integration of common features and complementary features. Consequently, for the image features ${F^{\prime }}_{ir}$ and ${F^{\prime }}_{vis}$ output from the MGRB module, the complementary features embedded within the visible features $Q_{vis}^{c}$, as well as those inherent in the infrared features $Q_{ir}^{c}$, can be obtained, which are defined as

(8) $$Q_{vis}^{c}={F^{\prime }}_{vis}-{F^{\prime }}_{ir},Q_{ir}^{c}={F^{\prime }}_{ir}-{F^{\prime }}_{vis}$$

The CAFB module is designed to fully exploit the complementary information across the dual branch features to realize feature interaction and pre-fusion. For a more precise definition, this study selects only the positive part of the complementary features as the supplementary information, which is expressed as follows:

(9) $$Q_{vis}^{c}=\left\{ \begin{array}{ll} Q_{vis}^{c}, & Q_{vis,ij}>0\\ 0, & otherwise \end{array}\right.,\;\;\;Q_{ir}^{c}=\left\{ \begin{array}{ll} Q_{ir}^{c}, & Q_{ir,ij}>0\\ 0, & otherwise \end{array}\right.$$

where $Q_{vis,ij}$ and $Q_{ir,ij}$ denote the values of $Q_{vis}^{c}$ and $Q_{ir}^{c}$ at point (i,j).

The weighted refined complementary features are acquired using CA and SA, respectively. For $Q_{vis}^{c}$, average pooling (AVGP) and max pooling (MAXP) operations are first performed on the spatial locations, and then the resulting features are concatenated along the channel dimension. The spatial weight map is generated after dimensionality reduction using 5 × 5 convolution operation and activation via the Sigmoid function. Lastly, the refined complementary feature $Q_{vis}^{1}$ with spatial location weights is derived by multiplying it with the input feature $Q_{vis}^{c}$. The computational process is represented as

(10) $$Q_{vis}^{1}=Q_{vis}^{c}⊗Sigmoid\left(Conv_{5}\left(C\left(AVGP\left(Q_{vis}^{c}\right),MAXP\left(Q_{vis}^{c}\right)\right)\right)\right)$$

For the CA stream, AVGP and MAXP are performed in the channel dimension followed by feature summation. The other operations are nearly identical to those of the SA stream, in addition to replacing the convolutional layer with two fully connected layers. A complementary feature, $Q_{vis}^{2}$, with channel weights is ultimately yielded. The calculation process is as follows:

(11) $$Q_{vis}^{2}=Q_{vis}^{c}⊗Sigmoid\left(FC_{2}\left(\sigma \left(FC_{1}\left(AVGP\left(Q_{vis}^{c}\right)⊕MAXP\left(Q_{vis}^{c}\right)\right)\right)\right)\right)$$

where $FC_{1}$ and $FC_{2}$ represent the first and second fully connected layers. Adding $Q_{vis}^{1}$ and $Q_{vis}^{2}$ yields a weighted complementary feature, ${\hat{Q}}_{vis}^{c}$, which is summed with the input infrared feature ${F^{\prime }}_{ir}$ to obtain a pre-fused feature ${\tilde{F}}_{ir}$ that contains both common features and complementary information of two source images.

(12) $${\tilde{F}}_{ir}={F^{\prime }}_{ir}⊕\left(Q_{vis}^{1}⊕Q_{vis}^{2}\right)$$

Pre-fused features ${\tilde{F}}_{vis}$ on the visible image path can be derived through similar steps.

${\tilde{F}}_{ir}$ and ${\tilde{F}}_{vis}$ are transferred as the feature output of the CAFB module to the deep feature extraction branch of source images to realize the information interaction and pre-fusion of infrared and visible features.

### 3.2. Loss Function

To yield fused images characterized by abundant texture details, prominent infrared targets, and clear edge contours, and to realize an end-to-end training approach, this research integrates the content loss $L_{Content}$, structural similarity loss $L_{SSIM}$, and perceptual loss $L_{Per}$ between fused images $I_{f}$ and original images $I_{ir}$, $I_{vis}$. Consequently, a comprehensive total loss function $L_{total}$ is devised, formulated as follows:

(13) $$L_{total}=\alpha L_{Content}+\beta L_{SSIM}+\gamma L_{Per}$$

where α, β, and γ represent the weighting parameters for each loss component.

#### 3.2.1. Content Loss

Content loss enables the generated image to gain rich texture information and approximate pixel distribution from the source image, which includes two parts, pixel loss $L_{pixel}$ and gradient loss $L_{grad}$, and is defined as follows:

(14) $$L_{Content}=\lambda L_{pixel}+L_{grad}$$

where λ is the weighting coefficient of pixel loss.

$L_{pixel}$ keeps the created image consistent with source images at the pixel level, and $L_{grad}$ forces the fused result to preserve greater high-frequency information for sharper texture details. The maximum pixel and gradient values among the visible and infrared images are employed to participate in the calculation to achieve the optimal luminance distribution and sharper texture details. The two types of losses are given by

(15) $$L_{pixel}=\frac{1}{HW}{\left\|I_{f}-\max\left(I_{ir},I_{vis}\right)\right\|}_{1},\;L_{grad}=\frac{1}{HW}{\left\|\left|\nabla I_{f}\right|-\max\left(\left|\nabla I_{ir}\right|,\left|\nabla I_{vis}\right|\right)\right\|}_{1}$$

where W and H represent the image width and height, ${\left\|•\right\|}_{1}$ denotes the l1-norm, and ∇ represents the gradient operator.

#### 3.2.2. Structural Similarity Loss

To diminish the edge imbalance and distortion of fused images, structural similarity (SSIM) loss $L_{SSIM}$ between the source and fused image is calculated from three aspects: brightness, contrast, and structure. For any two images x and f, SSIM is computed as follows:

(16) $$SSIM\left(x,f\right)=\frac{\left(2\mu_{x}\mu_{f}+C_{1}\right)\left(2\sigma_{xf}+C_{2}\right)}{\left(\mu_{x}^{2}+\mu_{f}^{2}+C_{1}\right)\left(\sigma_{x}^{2}+\sigma_{f}^{2}+C_{2}\right)}$$

where $\mu_{x}$, $\mu_{f}$, $\sigma_{x}$, and $\sigma_{f}$ indicate the mean and standard deviation of image pixels, $\sigma_{xf}$ denotes covariance between two image pixels, and $C_{1}$ and $C_{2}$ are constants. Then, $L_{SSIM}$ is given by

(17) $$L_{SSIM}=1-\frac{1}{2}\left(SSIM\left(I_{ir},I_{f}\right)+SSIM\left(I_{vis},I_{f}\right)\right)$$

A smaller $L_{SSIM}$ indicates higher structural similarity and better fusion performance.

#### 3.2.3. Perceptual Loss

Perceptual loss is initially used for image style transformation and image super-resolution to ensure the output image resembles the source image, thus obtaining a more favorable visual effect. Similarly, applying the perceptual loss to the image fusion task could enhance the feature similarity between fused and source images. A simple and efficient VGG-16 model is chosen as the feature extraction network for calculating $L_{Per}$ to prevent information loss due to the over-extraction of features. Given that the VGG-16 network receives 3-channel images as input, the fusion image is copied three times, while the target image is composed of source images $I_{ir}$ and $I_{vis}$ and the adjusted image $I_{adj}$. $I_{adj}$ is computed as follows:

(18) $$I_{adj}=\frac{1}{2}\left(I_{ir}+I_{vis}\right)$$

The feature maps convolved and activated in the tenth and thirteenth layers of the VGG-16 network are selected to compute the perceptual loss.

(19) $$L_{per}=\sum_{n=10,13}\frac{1}{C_{n}H_{n}W_{n}}{\left\|F_{t}^{n}-F_{f}^{n}\right\|}_{2}^{2}$$

where *n* indicates the sequence number of VGG-16 convolutional layers, $F_{t}^{n}$ and $F_{f}^{n}$ denotes feature maps of the target image and fused image after the *n*th convolution, $C_{n}$, $W_{n}$, and $H_{n}$ are the channel number, width, and height of feature maps, respectively, and ${\left\|•\right\|}_{2}$ denotes the l2-norm.

## 4. Experimental Evaluation

Several experiments are performed to validate the fusion performance of MEEAFusion in this section. Thirteen state-of-the-art methods are picked for comparison, comprising two traditional methods, i.e., GTF [44] and MDLatLRR [45]; two AE-based methods, i.e., DenseFuse [11] and RFN-Nest [12]; one GAN-based method, i.e., FusionGAN [8]; three end-to-end methods, i.e., PMGI [13], FLFuse-Net [14], and CMRFusion [46]; one transformer-based method, i.e., DATFuse [32]; one task-driven fusion method, i.e., IRFS [28]; two general image fusion methods, i.e., U2Fusion [22] and multi-task semi-supervised learning-based fusion framework (MSLFusion) [47]; and one diffusion model-based method, i.e., DDFM [34]. The parameters in the various methods are rigorously consistent with those in the original article while carrying out the comparisons.

### 4.1. Experimental Configuration

#### 4.1.1. Datasets

The Multi-Spectral Road Scenarios (MSRS) [43] dataset, which comprises 1444 matched visible and infrared image pairs, is adopted to train MEEAFusion, and this study divides the image pairs into the training set and test set in a 3:1 ratio. Three datasets including MSRS, TNO [48], and RoadScene [22] are used to evaluate the fusion effect of MEEAFusion in the testing phase. Notably, when testing on the latter two datasets, original images are directly fed into the test network without any re-training and parameter tuning to investigate the generalization performance of the algorithm.

#### 4.1.2. Implementation Detail

During the 50 epochs of MEEAFusion training, eight groups of images are randomly selected and stochastically cropped to the size of 128 × 128 as a batch input at each iteration. The Adam optimizer is adopted to update network parameters, with the learning rate initially set to 1 × 10^−3^, which is kept constant until linearly decaying after 25 epochs. Loss function weights for each part are set to α=0.5, β=10, γ=1, and λ=8. MEEAFusion is executed on a single GPU (RTX 3090), and the code is realized with the help of PyTorch.

#### 4.1.3. Evaluation Indicator

This paper applies both qualitative and quantitative methods to evaluate the fusion effect. During the qualitative evaluation, the information richness, the clarity of edge contour, and the overall visual effect are mainly considered, which are normally intermingled with some subjective consciousness. It is hard for the eyes to distinguish the strengths and weaknesses of the fused images with minimal differences as well. Hence, a series of quantitative evaluation metrics have been proposed to assess the performance of fusion algorithms more fairly and accurately. This research chooses eight objective evaluation indicators: spatial frequency (SF) [49], average gradient (AG) [50], edge-information-based indicators (Q_abf_) [51], edge feature mutual information (FMI_edge_) [52], multiscale structural similarity (MS_SSIM) [53], the sum of correlation differences (SCD) [54], natural image quality evaluator (NIQE) [55], and perception-based image quality evaluator (PIQUE) [56]. Among them, smaller NIQE and PIQUE indicate superior fusion outcomes, while larger other metrics showed better results.

Since these objective indicators mainly measure a single aspect of the image and cannot thoroughly determine the fusion effect, this paper establishes an average ranking as the overall evaluation indicator, which is calculated by averaging the rankings of all the indicators and serves to comprehensively assess the fusion quality.

### 4.2. Test Results on MSRS

#### 4.2.1. Qualitative Comparison

**Figure 6:**
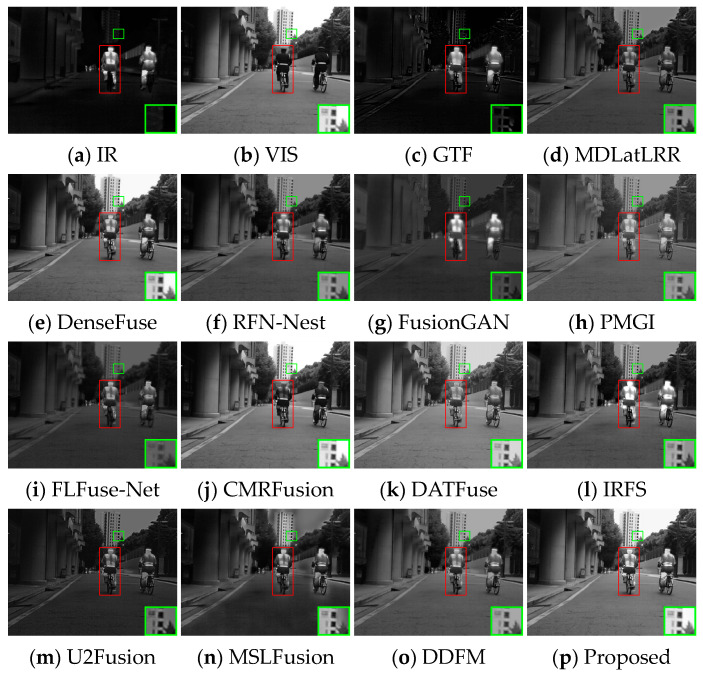
Visual display of fusion results for scene 00537D.

**Figure 7:**
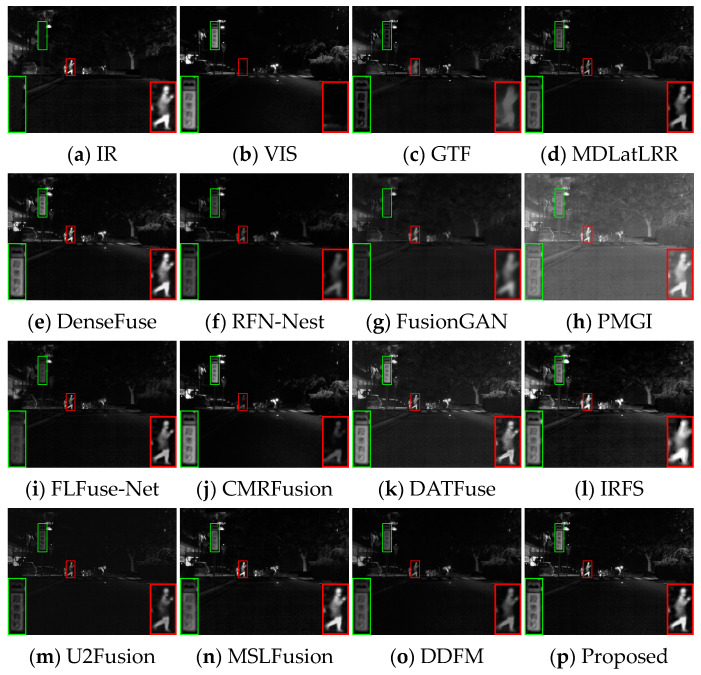
Visual display of fusion results for scene 00878N.

**Figure 8:**
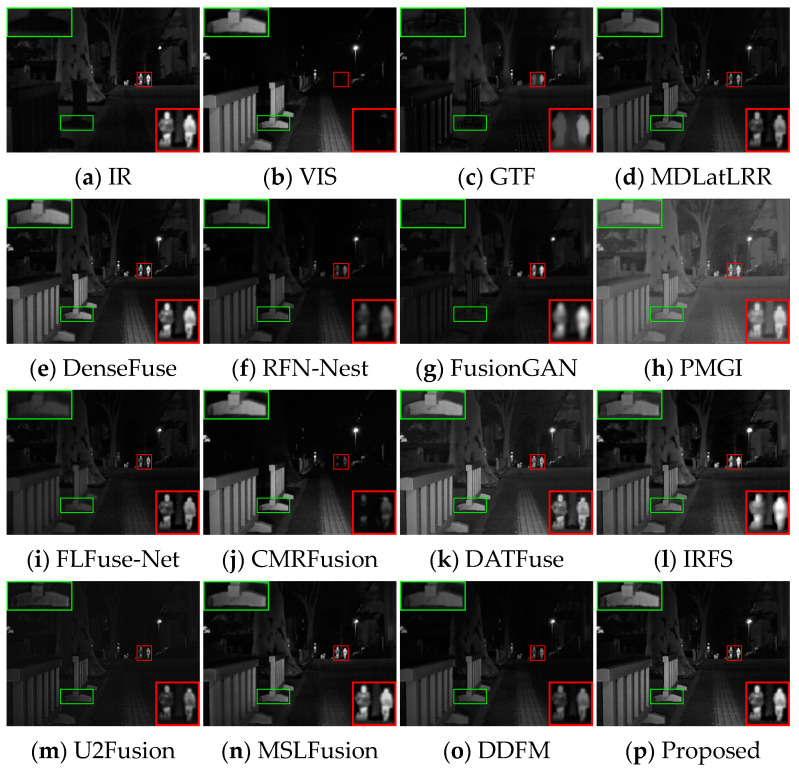
Visual display of fusion results for scene 01024N.

Several image pairs are selected in the MSRS dataset to visually compare the fused results yielded by different approaches, as presented in Figure 6, Figure 7 and Figure 8. The scenarios cover both daytime and nighttime conditions, and each scene image contains human significant targets and detailed regions. For clear comparisons, the salient and detailed regions are highlighted with red and green boxes, respectively, and the boxed regions are amplified in the corners of the images as needed.

Figure 6 illustrates that fused images of various algorithms present obvious differences in the daytime scene. GTF, FusionGAN, and IRFS have significant human targets (red boxes) but blurred details (green boxes). Among them, the GTF result is close to infrared images in terms of contrast with a severe loss of detail texture, the FusionGAN images have poorly defined edge contours, and the IRFS result is dark overall. The detail texture in MDLatLRR, DATFuse, and U2Fusion is relatively clear, but the infrared target is relatively weak. CMRFusion’s result exhibits a strong resemblance to the visible image, leading to a significant loss of infrared information. U2Fusion also lacks infrared features, appearing as image distortion. RFN-Nest, PMGI, FLFuse-Net, and DDFM have weak salient targets, and the image details appear less clear; the FLFuse-Net image especially encounters difficulty in distinguishing the leaves in the background. MSLFusion introduces spectral contamination and artifacts in the fused images. Only DenseFuse and the proposed method MEEAFusion could preserve infrared targets and background details better, and the fused image yielded by MEEAFusion has more salient targets and clearer edge contours thanks to the designed shallow multi-scale module, MGRB module, and CAFB module.

In the night scene, it is inaccurate to define IVIF narrowly as the preservation of background texture from visible images and salient targets from infrared images. This is because the imaging quality of the optical device is relatively inferior, while the infrared image contains the primary prominent target information, as well as the important background details. Therefore, the significant targets and the detailed texture in both images need to be considered simultaneously in this instance. As can be seen from Figure 7, GTF, RFN-Nest, and FusionGAN lose both salient targets (red box) and background texture information (green box), resulting in a devastating fusion effect. The images produced by MDLatLRR, FLFuse-Net, U2Fusion, and DDFM are similar to the weakened infrared images, exhibiting inconspicuous infrared targets and dark background information. CMRFusion’s result has a faint infrared target, while the IRFS image presents a distinct target but quite blurred edge contours. PMGI injects noise into the fused image, leading to poor image contrast. Figure 8 also exhibits similar fusion results. Only DenseFuse, DATFuse, MSLFusion, and the proposed method yield better fusion results with well-preserved infrared targets. However, MEEAFusion has the sharpest detailed texture, which can be attributed to the CAFB modules that fully preserve the complementary features in the visible and infrared images and consequently mitigate the interference of the illumination factors.

#### 4.2.2. Quantitative Comparison

**Figure 9:**
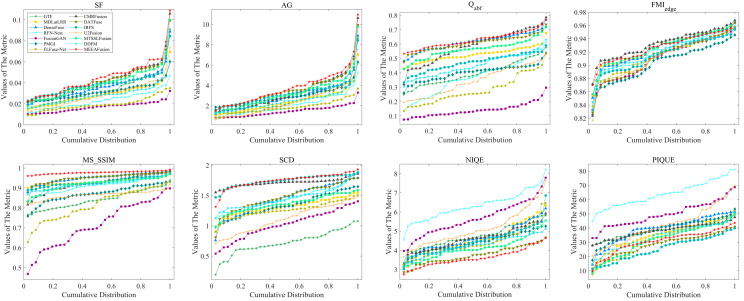
Data distribution of fusion results for 36 pairs of MSRS images over the eight objective evaluation criteria. Each point (x, y) in this Figure means (100 × x)% of fused images whose metric values do not exceed y.

From the MSRS dataset, 36 groups of images covering multiple scenes such as daytime and nighttime are randomly selected in this study. The calculated results of objective evaluation metrics are displayed in Figure 9 and Table 1. Notably, MEEAFusion manages to achieve six optimal results, among which the top SF and AG point out some improvement in the clarity and texture detail of MEEAFusion fused images. The maximum Q_abf_ illustrates that the presented method could retain more edge details from source images. The optimal MS_SSIM highlights the remarkable performance of the proposed approach in terms of contrast and brightness, while the best NIQE and SCD verify that MEEAFusion generates the highest-quality image that is closest to the original images. In addition, the second-best FMI_edge_ and the third-best PIQUE indicate higher mutual information and less image distortion. All of these excellent indicator values lead to the average ranking of MEEAFusion being first, indicating that the fusion results of MEEAFusion are superior in various aspects and have the best comprehensive quality.

Overall, the proposed method could effectively preserve the prominent targets and background textures in the original images, generating images with clear details, distinct edge contours, and optimal visual quality. This is mainly attributed to three factors: the shallow feature extraction module, the MGRB module, and the CAFB module. Certainly, it also benefits from the constraints of the loss functions of each part. These factors collectively facilitate the generation of high-quality fusion results.

### 4.3. Generalization Experiment

In the above comparative experiments, both training and testing of MEEAFusion are conducted on the same dataset. For validating the generalization performance of the proposed method, two additional datasets, TNO and Roadscene, are selected to further compare the fusion effects between multiple algorithms. A total of 20 and 30 image pairs containing different scenes are randomly picked from each dataset for subjective and objective evaluations, respectively.

#### 4.3.1. Test Results on TNO

**Figure 10:**
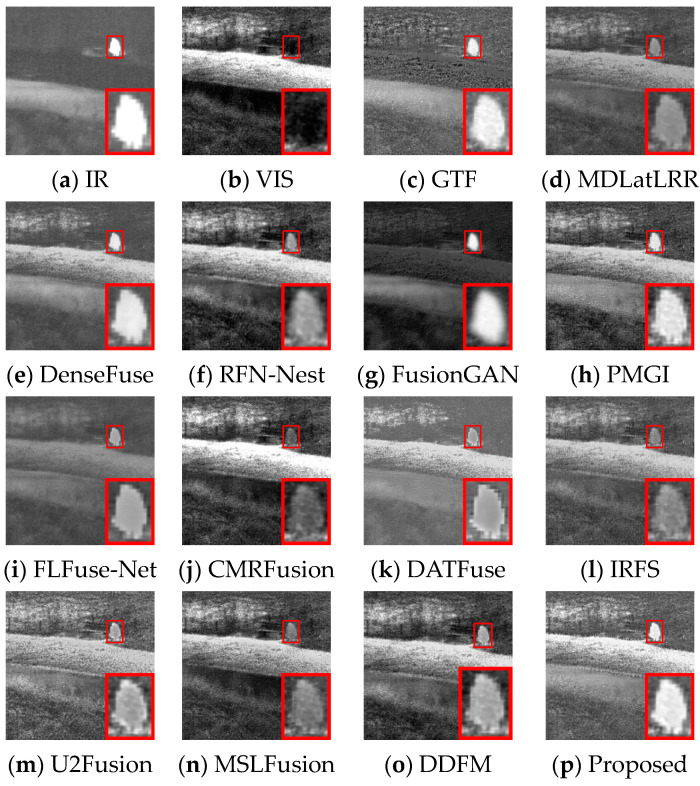
Visual display of fusion results for bench scene. The salient regions are highlighted with red boxes.

**Figure 11:**
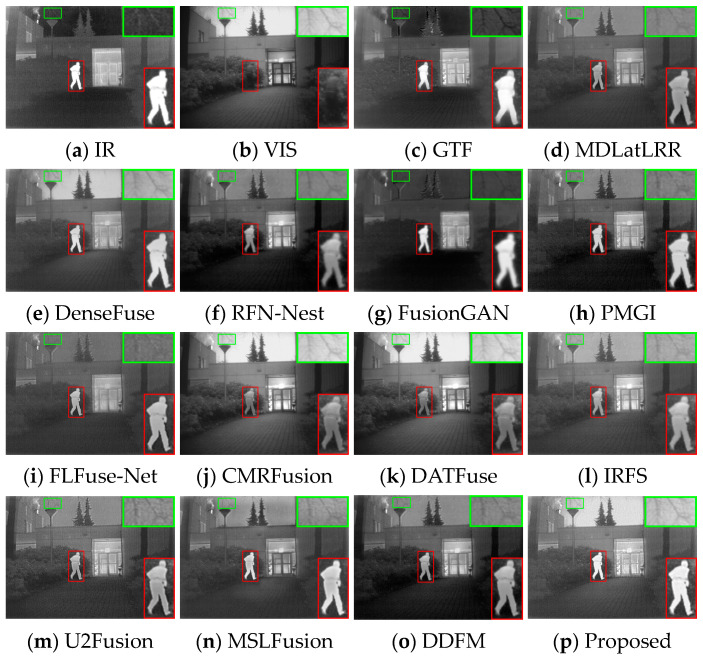
Visual display of fusion results for Kaptein_1123 scene. The salient and detailed regions are highlighted with red and green boxes.

**Qualitative Comparison.** Figure 10 and Figure 11 display two image pairs selected from TNO for visual presentation. As observed in Figure 10, all methods except GTF, DenseFuse, FusionGAN, PMGI, and the proposed method MEEAFusion, exhibit significant loss of infrared salient human targets. However, the fusion images of GTF and FusionGAN show imbalanced background information and unclear target edge contours. DenseFuse loses details, and the overall pattern becomes hazy. Only PMGI and MEEAFusion results demonstrate superior visualization with moderate global brightness, remarkable human targets, and sharp edge contours. Figure 11 further confirms the preceding results. MDLatLRR, PMG, U2Fusion, MSLFusion, and DDFM all produce darkened images with indistinct branch outlines. MSLFusion even exhibits light pollution. The results of DenseFuse, CMRFusion, DATFuse, IRFS, and MEEAFusion are more congruent with visual perception. Nevertheless, DenseFuse and MEEAFusion generate fusion images with brighter human targets and sharper human and tree branch outlines.

**Figure 12:**
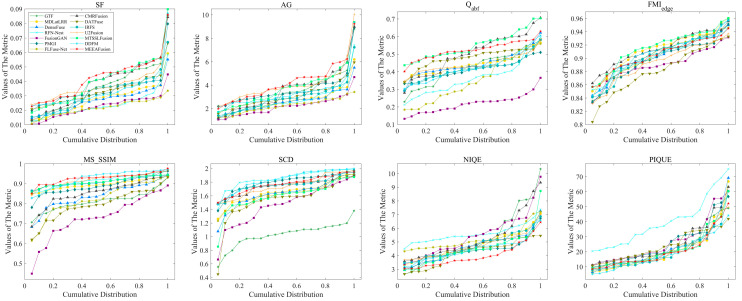
Data distribution of fusion results for 20 pairs of TNO images over the eight objective evaluation criteria. Each point (x, y) in this Figure means (100 × x)% of fused images whose metric values do not exceed y.

**Quantitative comparison.** Figure 12 and Table 2 demonstrate the objective evaluation results of MEEAFusion and the other 13 advanced algorithms on the TNO dataset. MEEAFusion achieves optimal results in four metrics, SF, AG, MS_SSIM, and NIQE, indicating that the method can obtain clearer images, richer texture information, higher structural consistency, and better visual presentation. The second-best result on Q_abf_ demonstrates that MEEAFusion retains more edge information. Although the results of FMI_edge_, SCD, and PIQUE perform slightly worse, they remain ranked at the forefront. Comprehensively, MEEAFusion ranks first, implying the approach suggested in this research has satisfactory generalization performance on an untrained TNO dataset.

#### 4.3.2. Test Results on RoadScene

**Figure 13:**
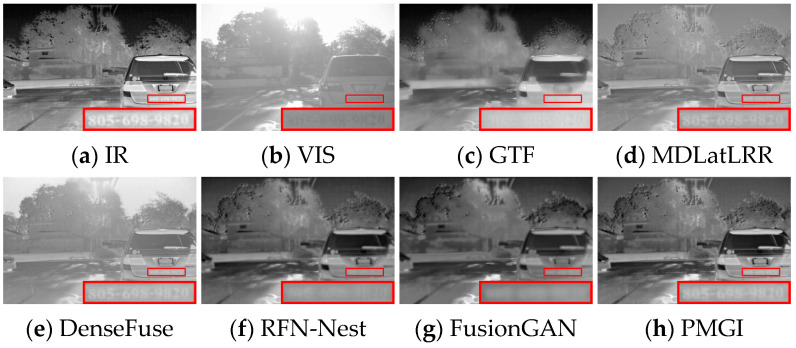

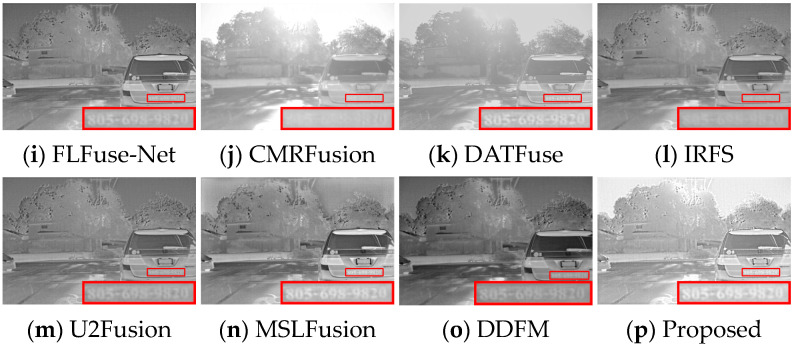
Visual display of fusion results for scene FLIR_00006. The detailed regions are highlighted with red boxes.

**Figure 14:**
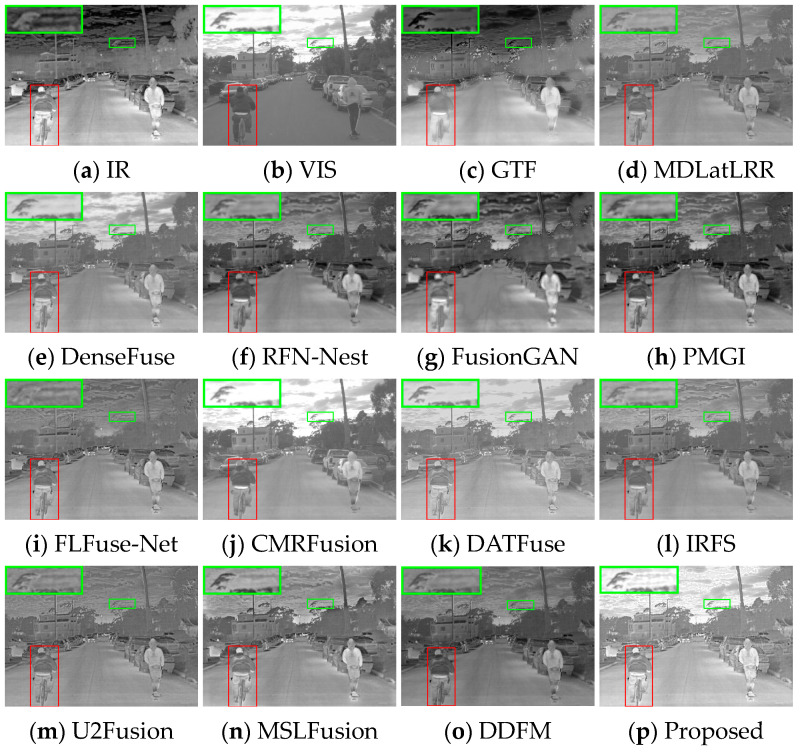
Visual display of fusion results for scene FLIR_06570. The salient and detailed regions are highlighted with red and green boxes.

**Qualitative Comparison.** Figure 13 depicts a visible image that appears to be overexposed due to strong sunlight. Confronted with this challenging scenario, GTF, RFN-Nest, FusionGAN, CMRFusion, and IRFS algorithms produce fusion results that suffer from a severe loss of infrared detail information and complete blurring of the license plate number. The fused images generated by MDLatLRR, PMGI, FLFuse-Net, U2Fusion, and DDFM are biased toward darkness, exhibiting low brightness in the detail regions. DATFuse fails to preserve the detailed texture of the overexposed region. Only MSLFusion and MEEAFusion manage to better retain the texture information of the overexposed region and infrared details at the same time. In Figure 14, the performance of the five methods such as GTF is far from satisfactory. The fused images continue to show significant target edge blurring, image distortion, and spectral contamination. Five approaches such as MDLatLRR over-preserve infrared detail information, resulting in low image contrast. DenseFuse, MSLFusion, and MEEAFusion retain texture details clearly and have better visual effects.

**Figure 15:**
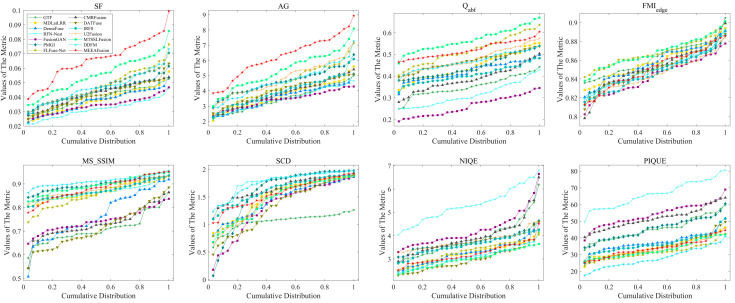
Data distribution of fusion results for 30 pairs of RoadScene images over the eight objective evaluation criteria. Each point (x, y) in this Figure means (100 × x)% of fused images whose metric values do not exceed y.

**Quantitative Comparison.** Figure 15 and Table 3 present the objective evaluation results of all methods on the RoadScene datasets. MEEAFusion exhibits a distinct advantage in SF and AG metrics, with values 18.61% and 16.72% higher than the second-placed method, respectively. This indicates that the produced images of MEEAFusion are clearer and richer in texture. The second-best Q_abf_ demonstrates that MEEAFusion is capable of retaining more edge information. The values of MS_SSIM, NIQE, and PIQUE rank at the forefront, implying that the fused images have a higher overall visual quality. Collectively, MEEAFusion ranks second, with slightly inferior fusion results than those on the TNO dataset. The reason is that the scene images in the TNO dataset are simpler and have higher contrast, while the RoadScene dataset images are more complex and include a variety of road and traffic scenes. Furthermore, the most probable explanation for MSLFusion ranking first is that MSLFusion is trained on both the TNO and RoadScene datasets, whereas the approach provided in this research is trained on the MSRS dataset and then evaluated straight on the RoadScene dataset without any parameter fine-tuning.

The aforementioned experimental results of qualitative and quantitative comparisons indicate that MEEAFusion still generates fusion images with significant targets and clear textures on untrained datasets, verifying that the proposed approach has outstanding generalization performance.

### 4.4. Efficiency Evaluation

Efficiency also plays an essential role in fusion performance evaluation. For a more fair and accurate evaluation of fusion efficiency, this paper fuses only one pair of source images every time by default. The fusion time is considered the duration from the input of the original images to the fusion system to the output of the fused image. This metric excludes the process of reading and saving the image, thereby comparing purely the run speed of the fusion network. The fusion efficiency is defined as the average fusion time of all image fusion pairs. Furthermore, all DL-based methods are run on a single RTX 3090 GPU, and the MATLAB code is executed on a single 2.60 GHz Intel i5-13500H CPU. 

Table 4 displays the parameter counts and inference time obtained for the different fusion approaches. The traditional methods take a longer time, whereas the fusion efficiency of the DL-based methods is dramatically improved due to the development of GPUs. FLFuse-Net exhibits the minimum parameter and fastest speed in the inference process. Noticeably DDFM employs 100 iterations of sampling in the process of generating the final fused image and, consequently, runs at the slowest speed, which hinders it from working in fields where real-time performance is required. The MEEAFusion algorithm in this study has relatively few parameters, which allows for its deployment on devices with more limited memory. It is observed that discarding dense connections could reduce the parameter numbers by 21.1% and lower the memory occupancy while boosting the running efficiency of the algorithm. The CAFB module slows down the inference speed due to the presence of fully connected layers. Nevertheless, MEEAFusion also possesses fusion efficiency comparable to that of other algorithms, maintaining excellent real-time performance on the trained MSRS dataset. What is more valuable is that the experimental results, both qualitative analysis and quantitative evaluation, demonstrate that MEEAFusion could yield fused images with better quality.

### 4.5. Ablation Experiment

**Figure 16:**
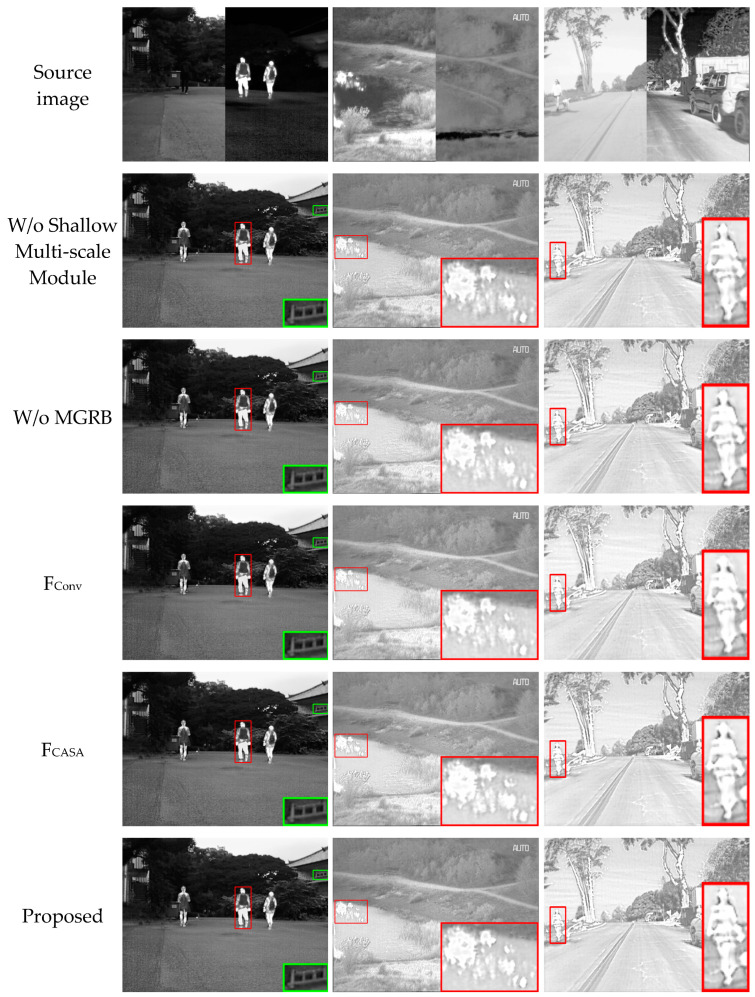
Visual results of the ablation experiment. The salient and detailed regions are highlighted with red and green boxes.

For the methods proposed in this paper, the shallow feature extraction modules, MGRB module, and CAFB module are the most critical components of the fusion network. Ablation experiments are performed to confirm the effectiveness of each module, and the depth of the network is guaranteed to remain constant during the ablation process. The experimental outcomes are displayed in Figure 16. 

Among them, the shallow multi-scale feature extraction module is replaced with a single 3 × 3 convolution, while the MGRB module removes the residual gradient stream and retains only the convolution operation on the mainline for deep feature extraction. Two approaches without feature interaction are adopted as alternatives to the CAFB module. One is F_Conv_, which employs normal convolution operations, and the other is F_CASA_, which still uses SA and CA but concentrates solely on the respective source image features. It can be found that without the shallow feature extraction module, the fused image produced by MEEAFusion has inconspicuous edge contours that can hardly be distinguished from the background. Additionally, the partial information loss leads to a decrease in brightness and the blurring of significant targets. The reason is that the shallow feature extraction module can provide informative multi-scale features for the deep feature extraction network and the image reconstruction module. Without MGRB, the fusion results show hazy edge contours of the salient target, unclear detail texture, and more image artifacts, which verifies that MGRB using multiscale gradient convolution could acquire deep features with enhanced edge information. The brightness, edge details, and contrast of the fused image degrade without CAFB, due to the loss of complementary feature information from different paths. F_CASA_ compels the IR and visible paths to pay more attention to their respective features through the attention mechanism, resulting in a slight improvement in fusion performance compared to F_Conv_. However, this also causes the redundancy of common features and spectral pollution. Only MEEAFusion preserves both clear texture details and salient infrared targets, thereby achieving a balance between infrared and visible information.

A quantitative comparison of the average rankings results of the ablation experiments is given in Table 5. It can be seen that the proposed algorithm achieves the best ranking on all three datasets. The worst results of the fusion algorithm on the MSRS and RoadScene datasets occurred when losing the MGRB module, which illustrates that the MGRB module dramatically improved the quality of the fused images in complex scenes. The numerical results on the TNO dataset remained unchanged, which is probably due to the simplicity of the image scenes. Without the CAFB module, the ranking metrics all undergo a drop; in particular, the F_Conv_ fusion results are noticeably worse than those of F_CASA_, which also further verifies the significance of the attention mechanism in image fusion.

### 4.6. Expansion to Object Detection Tasks

**Figure 17:**
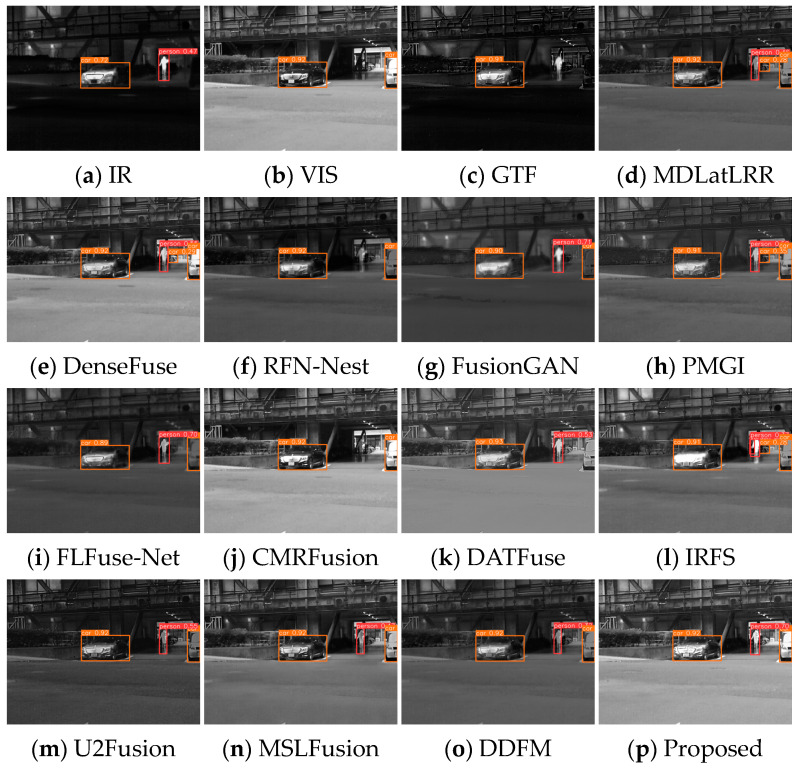
Visual display of YOLOv5s prediction results for fused images of scene 00479D.

**Figure 18:**
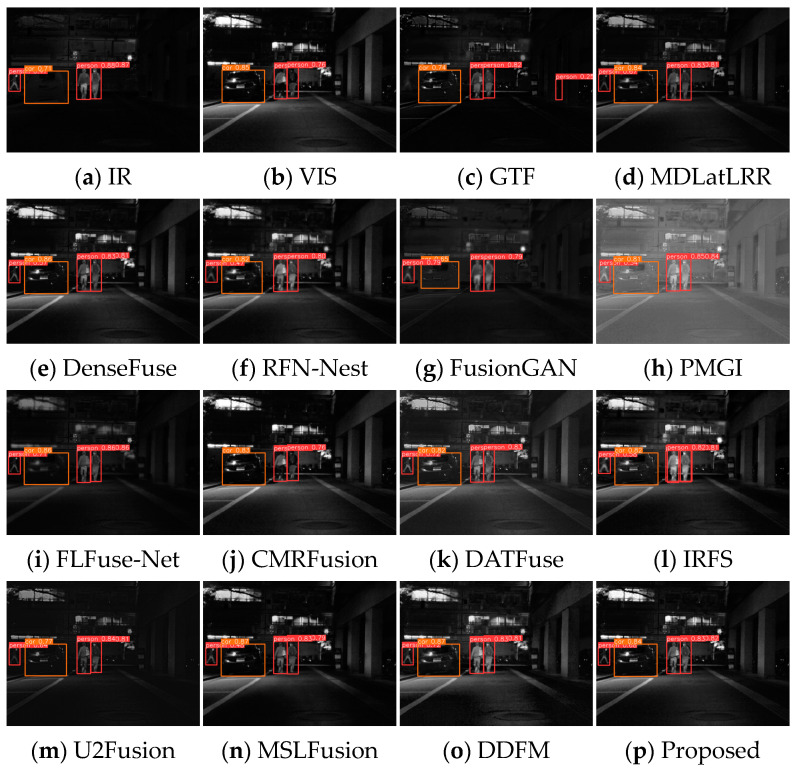
Visual display of YOLOv5s prediction results for fused images of scene 01348N.

To assess the improvement of image fusion processing on downstream advanced task results, the pre-trained YOLOv5s [57] algorithm is utilized to perform target detection on the fused images. Eighty pairs of images are randomly picked from the MSRS dataset as a test set, containing a variety of urban scene images, which are tagged as persons and cars. Figure 17 and Figure 18 show the prediction results of the fused images.

As illustrated in Figure 17, in the daytime scene, the detection result of the infrared image misses the vehicle. The human target in the visible light image is in the shadows and difficult to distinguish and also appears to be missed. The GTF image is close to the infrared image, but the details are more blurred, resulting in missed detection of both human and vehicle targets. The detection results of RFN-Nest, CMRFusion, and DATFuse lose either the person or car. Moreover, MDLatLRR, DenseFuse, PMGI, and IRFS show false detections. FusionGAN, FLFuse-Net, U2Fusion, MSLFusion, DDFM, and MEEAFusion correctly discover all persons and cars. In the night scenario, the infrared image results successfully detect all targets, and the visible image results miss the leftmost human target in the dark, as shown in Figure 18. Apart from GTF and CMRFusion, all other fusion algorithms could detect all objects correctly, verifying the facilitating function of image fusion processing for target detection.

The quantitative detection results of YOLOv5s on the MSRS dataset are shown in Table 6. Indicators of precision rate (P), recall rate (R), average precision (mAP), and average ranking are adopted to evaluate the detection results. As can be seen, the infrared image detection results are slightly better than the visible image results, but both source images have unsatisfactory detection precision. After image fusion, all fusion algorithm results, except GTF, achieve improved accuracy, indicating that the fused image could integrate the salient targets and detailed textures between the two source images and provide semantically rich input data for target detection. Among them, U2Fusion, MEEAFusion, and PMGI obtained the best detection results. The mAP@0.5 values of the detection results of fusion image produced by MEEAFusion are improved by 13.5% and 19.3% relative to that of the infrared and visible images, respectively. This indicates that the fusion process enhances the complementary semantic information, thereby improving target detection accuracy. In particular, the suboptimal mAP@0.5:0.95 indicates that the detection results of MEEAFusion fusion images have higher confidence.

## 5. Conclusions

This study introduces MEEAFusion, an innovative IVIF method leveraging multiscale edge enhancement and a joint attention mechanism. Two primary modules, MGRB and CAFB, have been devised. Firstly, the shallow multi-scale module is exploited to extract the unique feature information of the source images. Then, the MGRB module enhances the edge texture of the deep feature map by collecting the gradient information at different scales. Finally, after defining the complementary features between the visible and infrared images, the CAFB module facilitates the information interaction between the two source image features while diminishing the feature redundancy. The subjective and objective evaluation results demonstrate that MEEAFusion has promising generalization performance and could yield fused images with more prominent infrared targets and clearer image textures compared to state-of-the-art algorithms with comparable fusion efficiencies. Moreover, the fusion results of the proposed method can facilitate the detection performance. However, it can be observed that although MEEAFusion can fulfill the fusion task well under low light, the fusion performance declines under strong exposure conditions. Further exploration of achieving balance under diverse lighting conditions is essential in the future. Additionally considering integrating MEEAFusion with advanced vision tasks to fully maximize the superiority of image fusion processing is another future investigation direction.

## Figures and Tables

**Table 1:** Mean metrics values for 36 sets of MSRS fusion images. Bolded: optimal. Red and underlined: second best. Blue and italicized: third best.

Method	Year	SF	AG	Q_abf_	FMI_edge_	MS_SSIM	SCD	NIQE	PIQUE	Avg_Rank
GTF	2016	0.03390	2.62158	0.39717	0.91172	0.84263	0.72466	4.75139	38.51693	10.38
MDLatLRR	2020	0.02908	2.47235	0.52722	0.92621	0.93329	1.31517	4.59061	35.30279	7.25
DenseFuse	2019	0.03666	3.00071	0.61621	0.92546	0.95126	1.44971	4.61783	39.43270	6.13
RFN-Nest	2021	0.02335	2.08065	0.37068	0.91971	0.91265	1.45724	6.33071	63.94360	10.50
FsionGAN	2019	0.01703	1.46829	0.14063	0.91023	0.71779	1.02142	5.65274	49.62307	13.50
PMGI	2020	0.03179	2.91969	0.40145	0.90284	0.87247	1.34139	4.40355	38.90654	9.13
FLFuse-Net	2022	0.01958	1.86261	0.28108	0.91423	0.81813	1.26137	4.3925	31.98617	10.25
CMRFsion	2023	*0.04183*	*3.36720*	0.58690	**0.93255**	0.92485	*1.71234*	4.61313	38.15723	*4.63*
DATFuse	2023	0.04242	3.58402	*0.60683*	0.91174	*0.94985*	1.47268	3.74836	28.47054	3.63
IRFS	2023	0.03890	3.23311	0.47215	0.91485	0.93126	1.74468	4.47378	**25.62720**	5.00
U2Fusion	2022	0.02900	2.39662	0.34512	0.91206	0.86495	1.11638	5.13737	41.91133	11.25
MSLFusion	2024	0.03997	3.26022	0.56527	*0.92660*	0.92146	1.36440	*4.10945*	31.72690	4.88
DDFM	2023	0.02839	2.51313	0.45342	0.91922	0.92171	1.46218	4.24166	34.16000	7.13
Proposed	-	**0.04523**	**3.83346**	**0.62987**	0.92759	**0.97739**	**1.74649**	**3.60992**	*30.88926*	**1.38**

**Table 2:** Mean metrics values for 20 sets of TNO fusion images. Bolded: optimal. Red and underlined: second best. Blue and italicized: third best.

Method	Year	SF	AG	Q_abf_	FMI_edge_	MS_SSIM	SCD	NIQE	PIQUE	Avg_Rank
GTF	2016	0.03580	3.34028	0.42496	0.90426	0.81406	1.02760	5.19974	22.41869	9.25
MDLatLRR	2020	0.02766	2.64889	0.44399	*0.91325*	0.89546	1.65808	4.84399	21.28489	7.38
DenseFuse	2019	0.02720	2.60648	0.44623	0.90431	0.84084	1.63246	4.65275	22.38950	8.50
RFN-Nest	2021	0.02429	2.76698	0.36485	0.90342	0.91568	**1.84967**	5.61304	39.78446	9.50
FsionGAN	2019	0.02262	2.20098	0.21902	0.88986	0.73085	1.47160	5.48494	26.85803	13.38
PMGI	2020	0.03436	3.61049	0.41170	0.89793	0.89043	*1.79562*	*4.38430*	25.38754	7.13
FLFuse-Net	2022	0.02169	2.23774	0.35378	0.90487	0.82465	1.62571	5.21185	21.62141	10.50
CMRFsion	2023	*0.03847*	3.61020	*0.50599*	0.91449	0.85665	1.73990	5.32664	25.55392	6.63
DATFuse	2023	0.03345	3.10155	0.48017	0.88139	0.79700	1.53766	4.22719	24.05444	8.88
IRFS	2023	0.03265	2.95874	0.41742	0.90318	0.90902	1.7562	4.75725	20.42259	7.38
U2Fusion	2022	0.03798	4.08810	0.44275	0.89339	*0.92137*	1.74555	4.73371	*20.46969*	5.50
MSLFusion	2024	0.04063	*3.82522*	**0.55064**	**0.91677**	0.91071	1.59441	4.51435	21.77276	*4.38*
DDFM	2023	0.03380	*3.38253*	0.42670	0.90663	0.92628	1.83825	4.47876	**18.91701**	4.25
Proposed	-	**0.04247**	**4.25061**	0.52384	0.90528	**0.92783**	1.77066	**4.01078**	20.59162	**2.38**

**Table 3:** Mean metrics values for 30 sets of RoadScene fusion images. Bolded: optimal. Red and underlined: second best. Blue and italicized: third best.

Method	Year	SF	AG	Q_abf_	FMI_edge_	MS_SSIM	SCD	NIQE	PIQUE	Avg_Rank
GTF	2016	0.03926	3.50515	0.35250	*0.86218*	0.71048	1.05915	3.87581	45.53223	10.75
MDLatLRR	2020	0.03770	3.72310	0.44545	0.85838	0.86904	1.53087	3.23434	34.55284	7.25
DenseFuse	2019	0.03716	3.56279	0.42348	0.85754	0.76863	1.45553	3.28676	37.68914	9.13
RFN-Nest	2021	0.03181	3.46878	0.31513	0.85247	0.86711	**1.78102**	5.31723	66.31319	10.50
FsionGAN	2019	0.03435	3.43182	0.26227	0.84284	0.74475	1.32598	4.24944	54.06150	13.13
PMGI	2020	0.04387	4.60669	0.44314	0.85008	0.89718	1.58711	3.74076	45.66224	7.13
FLFuse-Net	2022	0.04622	4.48103	*0.52047*	0.86790	0.85427	1.49955	3.31725	31.48138	5.25
CMRFsion	2023	0.04240	3.84838	0.38696	0.85273	0.73314	*1.66402*	3.96085	51.73881	9.13
DATFuse	2023	0.04516	4.00403	0.46850	0.85103	0.71484	1.33434	**3.00270**	34.53270	7.38
IRFS	2023	0.04132	3.98069	0.40656	0.84701	0.87158	1.63911	3.59913	36.68563	8.13
U2Fusion	2022	0.04662	*4.82489*	0.47759	0.84525	0.87879	1.46428	3.77208	33.52812	6.63
MSLFusion	2024	0.05563	5.36458	**0.56856**	**0.86829**	*0.89578*	1.47067	3.00550	*32.96829*	**2.88**
DDFM	2023	*0.04690*	4.65157	0.46816	0.84995	**0.91240**	1.77356	*3.10277*	**28.02609**	3.88
Proposed	-	**0.06598**	**6.26128**	0.52271	0.85145	0.88223	1.57821	3.22964	33.99325	3.38

**Table 4:** Parameter numbers and inference time (s) for different fusion methods. Bolded: optimal.

Method	Year	Parameters (M)	RoadScene	TNO	MSRS
DL-based approach, computed on RTX 3090 GPU					
DenseFuse	2019	0.2226	0.0225	0.0227	0.0229
RFN-Nest	2021	7.5244	0.0364	0.0746	0.0837
FusionGAN	2019	1.3264	0.4978	0.3546	0.0273
PMGI	2020	0.0424	0.1387	0.0753	0.0105
FLFuse-Net	2022	**0.0086**	**0.0019**	**0.0018**	**0.0013**
CMRFusion	2023	1.0350	0.0083	0.0067	0.0057
DATFuse	2023	0.0108	0.0146	0.0245	0.0239
IRFS	2023	0.2417	0.0999	0.0569	0.0015
U2Fusion	2022	0.6592	0.6237	0.4318	0.0290
MSLFusion	2024	1.8037	0.1329	0.2141	0.2191
DDFM	2023	552.81	30.8919	56.5170	60.7564
Proposed	-	0.4141	0.3716	0.2613	0.0062
W/o Dense connections	-	0.3267	0.2963	0.2357	0.0055
W/o CAFB	-	0.3885	0.3598	0.2649	0.0046
MATLAB-based conventional method, computed on Intel i5-13500H CPU					
GTF	2016	-	1.6985	2.8846	3.7202
MDLatLRR	2020	-	27.8939	49.2648	53.8416

**Table 5:** Quantitative comparison of average ranking results of ablation experiments. Bolded: optimal.

Method	Datasets		
	MSRS	TNO	RoadScene
Proposed	**2.00**	**2.** **50**	**2.57**
W/o MGRB	3.63	**2.50**	3.43
W/o Shallow Multi-scale Block	3.13	2.63	2.86
F_Conv_	3.50	3.86	3.29
F_CASA_	2.75	3.50	2.86

**Table 6:** Quantitative detection results of YOLOv5s on MSRS fused images. Bolded: optimal. Red and underlined: second best. Blue and italicized: third best.

Method	P	R	mAP		Avg_Rank
			@0.5	@0.5:0.95	
IR	0.882	0.689	0.816	0.571	13.25
VIS	0.869	0.710	0.776	0.514	15.00
GTF	0.868	0.676	0.803	0.559	15.00
MDLatLRR	0.897	*0.875*	0.921	0.682	6.50
DenseFuse	0.908	0.904	0.923	0.687	5.25
RFN-Nest	0.900	0.839	0.905	0.636	10.00
FsionGAN	0.834	0.832	0.887	0.640	12.00
PMGI	**0.943**	0.845	*0.926*	0.671	*4.50*
FLFuseNet	0.910	0.874	0.925	*0.693*	4.75
CMRFsion	0.882	0.736	0.812	0.542	13.25
DATFuse	*0.930*	0.849	0.919	0.667	6.75
IRFS	0.919	0.717	0.857	0.609	10.50
U2Fusion	0.911	**0.918**	**0.938**	**0.700**	**2.25**
MSLFusion	0.909	0.839	0.911	0.669	8.25
DDFM	0.928	0.851	0.930	0.669	4.75
Proposed	0.938	0.857	*0.926*	0.694	3.00

## Data Availability

The original contributions presented in this study are included in the article; further inquiries can be directed to the corresponding authors.
